# Colchicine may assist in reducing granulation tissue in junctional epidermolysis bullosa

**DOI:** 10.1016/j.ijwd.2016.04.001

**Published:** 2016-05-19

**Authors:** Minhee Kim, Swaranjali Jain, Adam G. Harris, Dedee F. Murrell

**Affiliations:** a Department of Dermatology, St George Hospital, Sydney, NSW, Australia; b University of New South Wales, Sydney, NSW, Australia

**Keywords:** anti-inflammatory, colchicine, granulation tissue, junctional epidermolysis bullosa

## Abstract

Epidermolysis bullosa (EB) is a rare, inherited blistering genodermatosis. Patients with junctional EB (JEB) due to LAMB3 mutations have widespread blisters and erosions of skin, mucosae, and nails, creating significant physical, emotional, and psychosocial burdens. Here we report the use of colchicine for ameliorating hypergranulating wounds in a 41-year-old female with JEB generalized intermediate. Her skin wounds and granulation tissue gradually exacerbated under silicone dressings such that she became profoundly anemic. Subsequently, she was commenced on colchicine 500 μg daily on the basis that it may inhibit cell proliferation and be anti-inflammatory. After a 6-month trial of colchicine, she had an objective and subjective improvement in her validated EB Disease Activity and Scarring Index activity and damage scores and Quality Of Life in EB score with less skin erosions, granulation tissue, and erythema. In addition, her anemia resolved. She denied any gastrointestinal side effects. The exact mechanism of colchicine in assisting reduction of the blistering, erosions, and granulation in JEB is unclear, but the anti-inflammatory and antimitotic properties of colchicine may be partially responsible for this process.

## Introduction

Epidermolysis bullosa (EB) is a rare, inherited blistering genodermatosis. The hallmark feature of EB is extreme skin fragility and recurrent vesiculobullous eruptions following mild mechanical injury. Based on the level within which blisters arise, EB is divided into 4 major subtypes named EB simplex (EBS), junctional EB (JEB), dystrophic EB (DEB), and Kindler syndrome caused by 18 different gene mutations (Fine et al., 2014). Patients with more severe types of EB such as JEB and recessive dystrophic EB (RDEB) have more widespread blisters and erosions. JEB generalized intermediate, formerly known as JEB non-Herlitz, follows an autosomal recessive pattern and is characterized by decreased laminin-332 or collagen XVII staining (Fine et al., 2014). Chronic cutaneous inflammation and erosions with bleeding are responsible for anemia of chronic disease (Hwang et al., 2015); iron deficiency anemia; severe burning pain; malnutrition; and recurrent cutaneous infections for these individuals, creating significant physical, emotional, and psychosocial burdens. So far, there are no treatments that reverse the manifestations of EB. Protection of EB wounds is aimed at addressing the symptoms and preventing further trauma and secondary bacterial infections using nonstick dressings. Here, we report a case of JEB that demonstrated a dramatic response to colchicine.

## Case report

The patient was a 41-year-old Caucasian female with JEB generalized intermediate (EB-registry #14) caused by two different LAMB3 mutations (R635X/1588delAG) and who had been generally well, with two full-term pregnancies (Nakano et al., 2002). She had successfully given birth to two unaffected children (Choi et al., 2011). Prior to the establishment of the National EB Dressing Scheme in Australia in 2009 for funding of dressings, she could not afford silicone nonstick dressings and only used gentian violet on her erosions, air dried after a bath, without dressings and had only a limited number of erosions, which were well controlled. After June 2010, she began using silicone dressings (Mepilex, Mepitel, Mepilex Lite, and Mepilex Transfer) under the scheme as recommended by the EB nurse at the scheme. Subsequently, she gradually developed exuberant granulation tissue under the dressings, and her skin failed to adapt to the changes, despite several different nonstick silicone–based dressings being tried. At various times, weaning of these dressings was attempted, but the granulation tissue did not heal as before with the gentian violet and stuck to her clothes.

Over the last 2 years, she developed multifactorial anemia (hemoglobin fell in from 122 g/L in February 2013 to 89 g/L in February 2014) resulting from iron deficiency and chronic inflammation, weight loss, and nutritional deficiency. She had multiple blood transfusions for the severe anemia, which kept recurring within a few weeks, and skin infections with methicillin sensitive staphylococcus aureus and Group B streptococcus requiring 4 units of packed red blood cell transfusion, iron infusion, and long-term intravenous antibiotic administration. She was investigated with upper endoscopy, colonoscopy, and a pill-cam study without an identifiable source of bleeding as the hemoglobin dropped more precipitously than previously. Hemolytic screen was also negative. It was concluded that her anemia most likely resulted from chronic inflammation of her skin, marked cutaneous erosions, and iron deficiency.

Despite having regular bleach and salt baths to prevent recurrent skin infections, she suffered from recurrent skin infections requiring repeated courses of antibiotics. In October 2014, she developed acute renal failure and delirium during one of these episodes of sepsis, from which she recovered. The decision was made to trial 500 μg oral colchicine on the basis that it might inhibit cell proliferation and reduce inflammation. It took 6 months of persuasion for her to agree to try it. Baseline laboratory work was performed, including complete blood count, electrolytes, urea, creatinine, liver function test, and C-reactive protein (CRP).

In our EB clinics, patients are routinely monitored using validated EB-specific outcome measures, the EB Disease Activity and Scarring Index (EBDASI) and the Quality of Life in EB (QOLEB) questionnaire. The EBDASI distinguishes activity scores separately from damage. The total activity score is 276, and a total damage score is 230 (Loh et al., 2014). The total score for the QOLEB is 51, and a lower score on the QOLEB correlates with the better quality of life (Frew et al., 2009). Her scores had been monitored during the previous year at each of her visits.

**Fig. 1:**
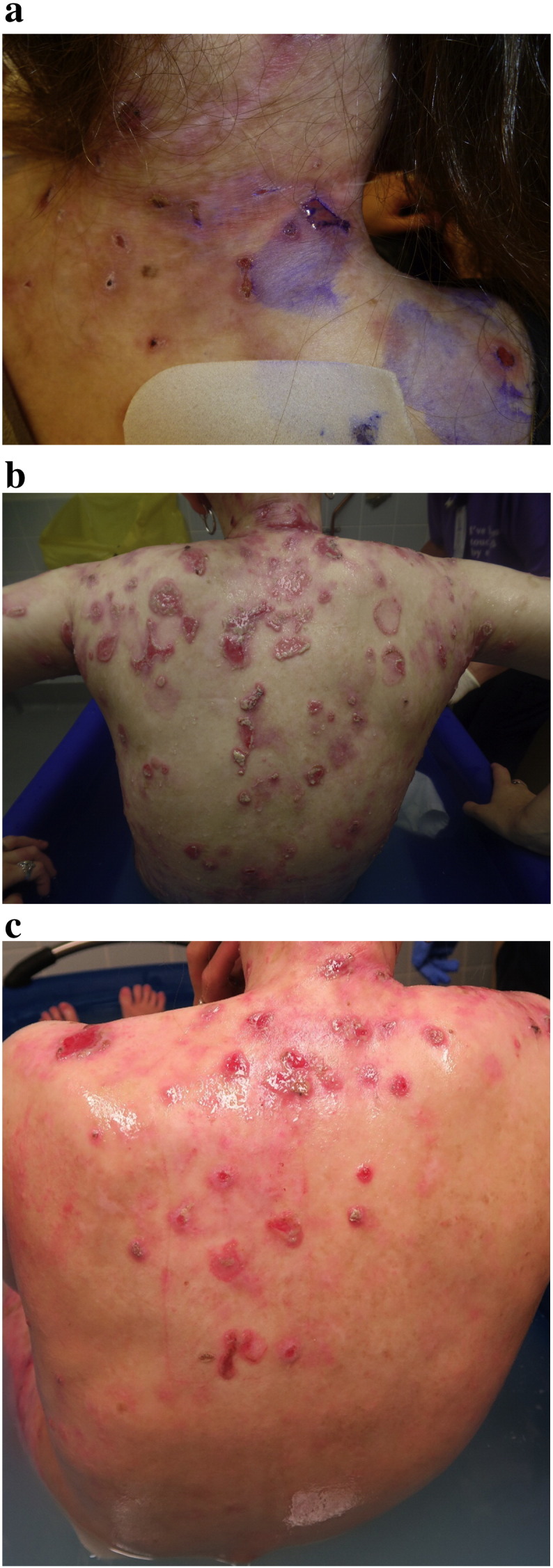
Clinical response to colchicine. a) Before using silicone dressings. Multiple skin erosions painted with gentian violet. No surrounding erythema or inflammation. b) Before the commencement of colchicine. Significant skin erosions with granulation tissue, surrounding erythema and inflammation. c) After taking colchicine for 6 months. Clinical improvement with fewer erosions and granulation tissue and erythema.

**Fig. 2:**
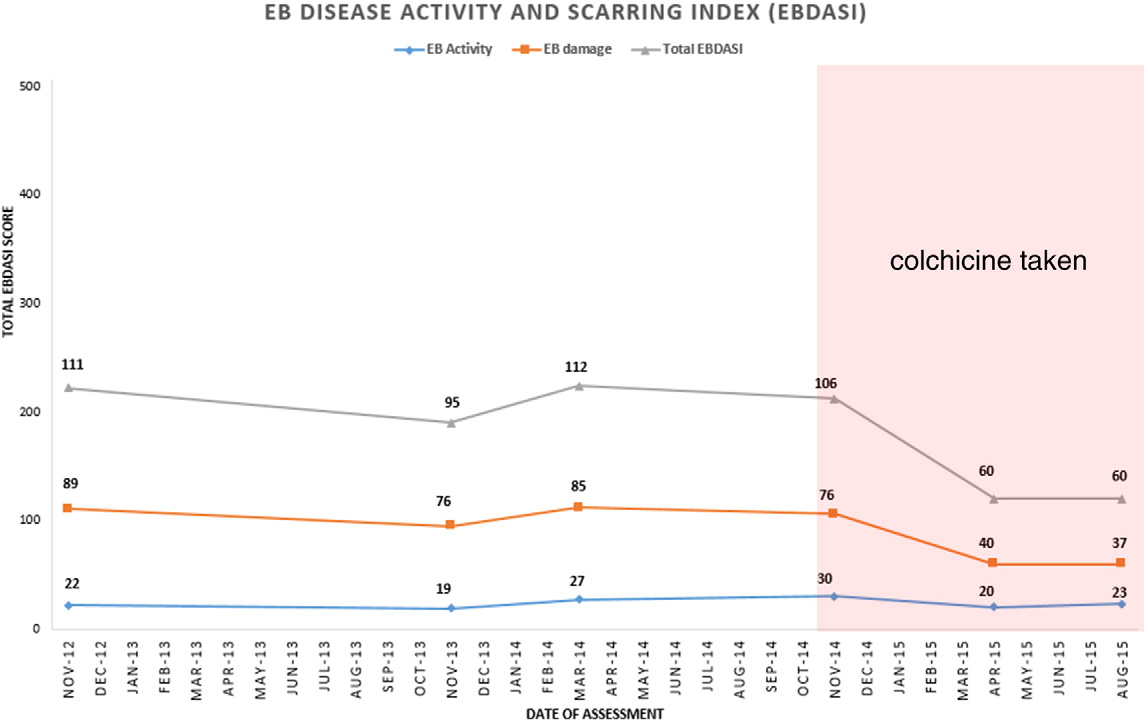
Epidermolysis Bullosa Disease Activity and Scarring Index (EBDASI) score with the pink area indicating the duration of colchicine intake. Improved EBDASI total score from 106 at the baseline to 60. EBDASI activity score improved from 30 to 23. EBDASI damage score improved from 76 to 37.

**Fig. 3:**
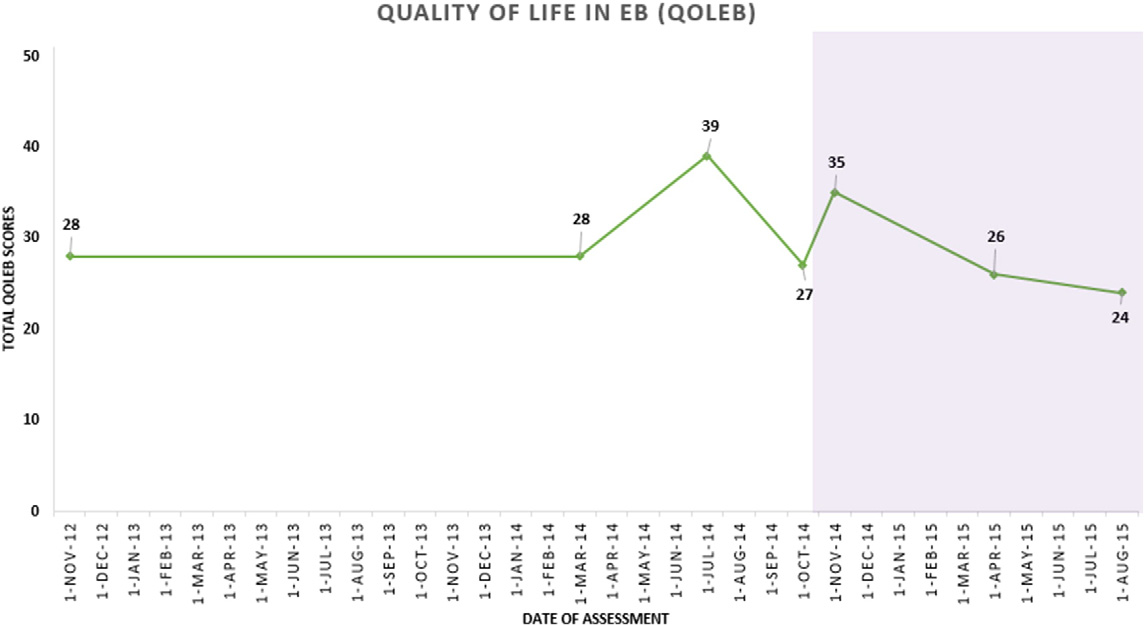
Quality of Life in Epidermolysis Bullosa (QOLEB) score with the purple area indicating the duration of colchicine intake. Improved quality of life evident by QOLEB scores from 35/51 at baseline to 24/51 after 6 months of colchicine treatment.

Three monthly blood testing was performed to monitor serological response to colchicine. This patient was followed up at 3 months during the 6-month period of colchicine therapy. Full skin examination was performed, each time with a complete removal of all the dressings in the designated EB bathroom. She felt symptomatically better after starting colchicine, with less stinging and burning sensation of the skin. She denied having gastrointestinal symptoms such as nausea, vomiting, or diarrhea. Clinically, there were fewer skin erosions and granulation tissue and erythema (Fig. 1, a–c). Her EBDASI score improved from 106 total (30 for activity and 76 for damage) at the baseline to 60 (23 for activity and 37) for damage after 6 months of colchicine treatment (Fig. 2). Her quality of life score, which had gradually worsened over the preceding few years, also improved from 35/51 to 24/51 after the 6 months of colchicine treatment (Fig. 3). Blood studies did not reveal any rare hematological side effects, including agranulocytosis, thrombocytopenia, and aplastic anemia. Renal function remained stable, and her hemoglobin improved significantly from 95 g/L at the baseline to 128 g/L after the 6 months. A steady decline of her elevated CRP level was also observed (Table 1).

## Discussion

Patients with JEB and some with recessive DEB have exuberant cutaneous granulation tissue resulting from repetitive phases of wound healing, including inflammation, re-epithelization, angiogenesis, and tissue remodeling. As mentioned in this case, a number of complications may arise from chronic erosions and inflammation. According to the US National EB Registry population, death from sepsis was more frequently seen in severe types of EB including JEB and RDEB. The cumulative risk of death from sepsis by age 60 was the highest, at 35.4%, in JEB non-Herlitz subtypes (Fine, 2009). Frequent hospitalizations, impaired wound healing, bacterial colonization of chronic nonhealing skin wounds, and recurrent use of topical and oral broad-spectrum antibiotics predispose these patients to sepsis with more resistant microorganisms. The prevalence of anemia in JEB and RDEB was much higher, at 68.8% and 68.0%, respectively, compared to that of EBS at 11.3% (Hwang et al., 2015).

Anemia in EB is thought to stem from chronic mucocutaneous inflammation, blood loss through skin wounds, and compromised bone marrow response to the elevated levels of erythropoietin. The onset of these problems after commencement of silicone-based dressings in this patient is concerning. There has been no randomized controlled trial to compare the efficacy and safety of these types of dressings in EB with more traditional nonstick dressings, such as Jelonet or Vaseline gauze. Silicone dressings when used for other conditions are usually left in place for up to a week for localized wounds. However, in EB, particularly in humid environments such as Australia, dressings need to be changed every 1 to 2 days to avoid the excess colonization of bacteria in wounds that occurs under occlusive dressings. The dressings are not cut to fit exactly into the wounds. They are square dressings, and the patients’ normal skin as well as the wound bed is in contact with the silicone surface. This particular patient has always taken baths on a daily basis and continued to do so even when the dressings were applied, because she did not like the odor under the dressings.

Colchicine is an alkaloid extracted from the plant *Colchicum antumnale* (Bibas et al., 2005). Anton Von Störck first used colchicine for the treatment of acute gout arthritis in 1763 (Ahern et al., 1987). It is well known for its antimitotic and anti-inflammatory properties. It interferes with microtubule formation by forming high-affinity complexes with dimers of tubulin and subsequently inhibiting the transformation of tubulin into microtubules. It is also thought to increase the activity of collagenase and decrease collagen production. For this reason, colchicine has been tried as an adjuvant therapy for treating keloid scars in small case studies and has shown some benefit (Singler, 2010).

Colchicine has been the standard treatment for gouty flares for some time. It is also widely used for the treatment of amyloidosis associated with familial Mediterranean fever. In dermatology, colchicine has been used in treating a number of neutrophilic dermatoses, including Sweet’s syndrome, dermatitis herpetiformis, psoriasis, and Behçet’s disease (Bibas et al., 2005).

Colchicine has also been used for some bullous and ulcerative disorders including EB acquisita (EBA) and ulcerative necrobiosis lipoidica (Cunningham et al., 1996, Schofield and Sladden, 2012). Cunningham et al. used colchicine in 4 patients with EBA refractory to conventional therapy and observed reduced blister formation and skin fragility. The study postulated that the neutrophil infiltrate in early EBA initiating the inflammatory cascade may have been retarded by colchicine by interfering with polymorphonuclear chemotaxis. Another case study reported on a patient with a 10-year history of bilateral ulcerative necrobiosis lipoidica on the shins who experienced a complete resolution after taking colchicine for 2 months (Schofield and Sladden, 2012). It was thought that colchicine may work by modulating inflammatory responses by inhibiting tumor necrosis factor alpha and the nuclear factor-kB signaling pathway. Granulation tissue in JEB consists of collections of mononuclear inflammatory cells, macrophages, neutrophils, and cellular debris. Hence, this aberrant manifestation of wound healing may have been reduced. Colchicine was tested in cultured explants from patients with EB simplex and DEB but not JEB, demonstrating an increase in collagenase expression (Bauer and Tabas, 1987).

## Conclusion

Use of colchicine to treat inflammation in EB wounds has not been reported in the literature, to our knowledge. The antimitotic and anti-inflammatory properties of colchicine suggest it has a potential role in JEB. Here we report a case where colchicine has improved skin healing, reduced skin inflammation, and improved anemic complications related to JEB. In addition, the extent of use of silicone based dressings for patients with limited junctional EB may need to be reviewed, particularly when they are applied to the normal skin around the wound for protection.

## Figures and Tables

**Table 1:** Blood parameters (colchicine commenced in November 2014). No evidence of hematological side effects. Improvement of hemoglobin with a steady decline in CRP.

Parameters	Oct 10, 2014	Oct 28, 2014	Nov 19, 2014	Feb 18, 2015	July 13, 2015	Normal range
Hb (g/L)	78	104	95	111	128	115-165
Platelets	459	443	492	432	550	150-450
WBCs	6.1	7.1	6.9	9.6	10.9	4-11
MCV	78	83	85	87	89	80-97
CRP	202.1	N/A	150.3	42.1	47.3	< 3

CRP, C-reactive protein; Hb, hemoglobin; MCV, mean corpuscular volume; WBCs, white blood cells.
